# Mixed Methods Approach to Describe Social Interaction During a Group Intervention for Adolescents With Autism Spectrum Disorders

**DOI:** 10.3389/fpsyg.2019.01158

**Published:** 2019-06-04

**Authors:** Carlota Alcover, Ma. Ángeles Mairena, Marcela Mezzatesta, Neus Elias, María Díez-Juan, Gemma Balañá, Mireia González-Rodríguez, Jairo Rodríguez-Medina, M. Teresa Anguera, Eulàlia Arias-Pujol

**Affiliations:** ^1^Faculty of Psychology, Education and Sports Sciences, Ramon Llull University, Barcelona, Spain; ^2^Children and Adolescent Mental Health Research Group, Institut de Recerca Sant Joan de Déu, Santa Rosa, Esplugues de Llobregat, Spain; ^3^Child and Adolescent Psychiatry and Psychology Department of Hospital Sant Joan de Déu of Barcelona, Passeig Sant Joan de Déu, Esplugues de Llobregat, Spain; ^4^Center for Transdisciplinary Research in Education, University of Valladolid, Valladolid, Spain; ^5^Faculty of Psychology, Institute of Neurosciences, University of Barcelona, Barcelona, Spain

**Keywords:** Qual-Quan integration, mixed methods, systematic observation, polar coordinate, social skills interventions, ASD, group

## Abstract

Over the last 20 years, researchers have been mixing qualitative and quantitative approaches, but mixed methods research represents a new movement that arose in response to the currents of qualitative and quantitative research, considered separately. Little has been published on the use of polar coordinate analysis in psychotherapy. This type of analysis can provide detailed information and integrate the qualitative-quantitative analysis. Even less has been published on the analysis of ASD children’s behavior. The main aim of this study was to implement this mixed methods methodology to analyze patterns of social behaviors in a group of adolescents with ASD during a group social competence intervention program. Moreover, we wanted to see whether an observational scale could be combined fruitfully with polar coordinate analysis and to investigate whether typical ASD behaviors show similar interrelations (prospective and retrospective sequentialities) as behaviors observed in psychotherapy. We used an adaptation from the Social Skills Training Program (UC Davis, California). We observed that each participant took a unique course, increasing or decreasing the number and quality of their social behaviors. In accordance with previous literature, results suggest some increment in the amount of appropriate social conduct. We did not detect a generalized progress pattern but agreed that there were changes between the beginning and end of the intervention. Therefore, we consider that observational methodology is useful in the field of psychotherapy and ASD, offering detailed information about changes and development that cannot be obtained with other traditional measures, such as questionnaires.

## Introduction

Autism spectrum disorders (ASD) are serious neurodevelopmental disorders that involve impairments in reciprocal social interaction and social communication combined with restrictive interests, repetitive behaviors and sensory abnormalities, and a wide range of psychiatric and medical conditions (American Psychiatric Association [APA], 2013). The estimated prevalence of ASD is about 1/160 (World Health Organization [WHO], 2017).

Regarding treatment, social skills training programs (SSTP) constitute an evidence-based therapeutic approach commonly used with high-functioning adolescents with ASD. Certain specific manualized programs have demonstrated positive results in improving social competence in adolescents with ASD (DeRosier et al., 2011; McMahon et al., 2013). However, it is difficult to adequately measure improvements in social skills. Most studies that evaluate the effectiveness of group therapies commonly use questionnaires or social cognitive assessments to measure social outcomes (Kasari et al., 2014) but these evaluations have been proven to be insufficiently sensitive and other methods of evaluating these outcomes are urgently required.

According to Sánchez-Algarra and Anguera (2013), systematic, objective, serious study of human conduct is highly complex due to the spontaneity of this behavior and the natural context of the situation. Considering this complexity, integration of qualitative methods, which provide a holistic view of the person, and quantitative methods, which offer more objective information (Lutz and Hill, 2009) has been proposed. It is for this reason that observational methodology is used in the field of psychology research. Observational methodology allows analysis of spontaneous behaviors that occur in the natural environment (Portell et al., 2015) and has been shown to be useful in studying changes that take place over the course of psychotherapy (Pascual-Leone et al., 2009). Moreover, this type of methodology can directly evaluate social performance and behaviors linked to the objectives of the intervention. For instance, McMahon et al. (2013) proposed the use of an observational scale (Bauminger, 2002) to evaluate the efficacy of a group-based intervention for children and adolescents with ASD.

Observational studies are a form of mixed methods research. After researchers had been more than 20 years mixing qualitative and quantitative approaches, mixed methods research is the response to the currents of qualitative and quantitative research, and represents a new movement that has arisen. Arias-Pujol and Anguera, 2017). The mixed methods approach involves the collection, analysis, and interpretation of qualitative and quantitative data for the same purpose and within the framework of the same study (Anguera et al., 2018). Studies that use observational methodology are considered to be mixed methods research studies, as they offer a great amount of data that can be analyzed in different ways. Data obtained from observational methodology are qualitative but can be transformed into quantitative data using different techniques, such as sequential analysis and polar coordinate analysis. These quantitative data can also be analyzed from a qualitative perspective. This integration of qualitative and quantitative methods is coherent with a mixed methods perspective (Anguera et al., 2017). In mixed methods studies we can perform various types of analysis. A study by Arias-Pujol et al. (2015) used lag sequential analysis to identify patterns of interactive behaviors between children with ASD and their therapists during psychotherapy sessions. Results show how certain behaviors exhibited by the therapist stimulated the appearance of positive behaviors in the child.

The analysis of polar coordinates is distinct from other forms of sequential analysis. Polar coordinate analysis reveals the relationships established between a behavior considered as “focal” and a number of conditional behaviors, with respect to prospective and retrospective sequentialities, and to describe different behavioral maps (Castellano and Hernández, 2003). This analysis provides information about activation or inhibition of the registered/observed behaviors through qualitative information transformed into quantitative data, allowing detailed observation of diverse behaviors.

Polar coordinate analysis is an elaborate data reduction technique that provides a vector image of the complex network of interrelationships between categories that make up the different dimensions of the observation instrument. The structure of polar coordinate analysis, which is a technique that complements prospective and retrospective sequential analysis (Bakeman, 1978), is based on complementarity between two analytical perspectives: prospective and retrospective. Therefore, this technique can detect changes in social competence behaviors in adolescents with ASD throughout therapy by combining data from prospective and retrospective viewpoints.

Little has been published on the use of polar coordinate analysis or similar analyses to examine the behavior of children (Herrero, 2000; Espinosa et al., 2004; Rodríguez-Medina et al., 2018). In ASD, the literature is even scarcer. Mixed methods analysis has been used to describe interactions in neurotypical adolescent group therapy (Arias-Pujol and Anguera, 2017). In their study, Arias and Anguera used polar coordinate analysis to analyze conversation turn-taking between therapists and six adolescents over the course of a 24-session intervention. Results show that polar coordinate analysis can offer a new focus. It could be used to study the role of the therapist, her interaction style and the effects of her strategies on participants.

In this study, we used an adapted version of the social competence intervention program developed by the Solomon Lab at the UC MIND Institute (Solomon et al., 2004). The goal of the group therapy is to improve social skills and social competence. Therapists seek to promote social interactions, such as conversation and cooperative play. Sessions include topics such as empathy, talking about feelings and resolving social conflicts.

The main aim of this study was to demonstrate how polar coordinate analysis can be useful in studying social behaviors in adolescents with ADS during an intervention. Moreover, we want to see whether Bauminger’s scale could be combined fruitfully with polar coordinate analysis and to investigate whether typical ASD behaviors show similar interrelations (prospective and retrospective sequentialities) as behaviors observed in psychotherapy.

## Materials and Methods

### Design

In this study, we applied systematic observation to analyze social behaviors in a group of adolescents with ASD. A total of ten sessions was conducted, although the first session was not included in the analysis as participants did not know each other and this could interfere in interactions. We only observed 15 min of each session (free play time). The observation of behavior was scientifically rigorous as the observers had a non-participatory role and only observable behavior was coded.

The observational design (Anguera et al., 2001) was nomothetic (several adolescents were observed), included follow-up (every session from the intervention was registered), and multidimensional (several dimensions of the observation instrument were considered suitable) (N/F/P). As the therapeutic process extended to several sessions, the group of adolescents was considered as a plurality of units. We worked with two levels of response: verbal and non-verbal.

### Participants

Following approval from the Research Committee Review Board at Sant Joan de Déu Hospital (Barcelona) and the Ethical Committee for Clinical Research at Sant Joan de Déu Foundation (CEIC “Comitè d’Ética d’Investigació Clínica Fundació Sant Joan de Déu”), subjects were recruited by psychologists and psychiatrists from the Multidisciplinary Autism Spectrum Disorder Unit (UnimTEA), Hospital Sant Joan de Déu (HSJD). All parents provided written, informed consent and informed assent was obtained from each child. Participants were informed about the location of the camera and the period of time that would be recorded.

Participants were selected through inclusion criteria based on an age range between 13 and 17 years old, diagnosed with Autism Spectrum Disorder according to DSM-5 criteria, and evaluated with the Autism Diagnostic Observational Schedule-2 (ADOS-2; Lord et al., 2000). Subjects were also required to have a level of Verbal Comprehension within the normal range according to standardized assessment. Participants with below average cognitive or language abilities (IQ < 70), severe behavioral problems, and/or other mental psychopathologies were excluded.

A total of eight adolescents were initially enrolled and participated in ten group sessions. However, the final sample consisted of five participants as three participants did not attend all sessions (mean age 14.6; 1 Girl/ 4 boys).

The group was led by a trained psychologist and assisted by co-therapists.

#### Intervention Design

The intervention was based on a more extensive program developed by the Solomon Lab at the UC MIND Institute (Solomon et al., 2004). The main goal of the program was to develop and improve social competence skills, following an “inside-out” model, that is, trying to increase inner motivation to socialize. Each session consisted of separate parts: greeting time, 15 min of free play, didactic time, joke time and a take-home social experiment. Didactic time involved training abilities such as empathy, recognizing emotions, managing anxiety or anger, reciprocal conversation skills, theory of mind and problem-solving.

The adolescents included were invited to participate in a total of 10 sessions, scheduled on a weekly basis. These sessions took place in a large, specially adapted room at Sant Joan de Déu Hospital (Barcelona) and lasted for 90 min. Each session followed the same structure: introduction (open conversation), 15 min of free play, didactic time, jokes and ending. Free play time in all sessions was video-recorded.

Before the group sessions, all participant families attended an information session with therapists and general information about the study was provided. Afterward, each participant had an individual meeting with his/her therapist to set his/her own personal goals.

Participants agreed to participate in the program and informed consent was obtained from the parents of minors. All procedures were in accordance with the ethical standards of the institutional research committee and the 2000 Declaration of Helsinki.

### Instruments

#### Diagnostic Instruments

Diagnosis of Autism Spectrum Disorder was confirmed through clinical interview and the ADOS-2 (Lord et al., 2000), which was administered to the adolescents. Cognitive abilities were measured with the Wechsler intelligence scale for children and adolescents: Fourth edition (Wechsler, 2007) or Fifth edition (Wechsler et al., 2014).

#### Recording Instrument

Group sessions were recorded using two video cameras. In accordance with the principles of the Declaration of Helsinki, the Spanish Official College of Psychologists General Council’s Ethical Code and the Ethical Committee for Clinical Research at Sant Joan de Déu Foundation, participants were informed that they were being filmed. They were shown the location of the video cameras, which were positioned discreetly to minimize reactivity bias. Lince software (Gabin et al., 2012) was used to codify behaviors.

#### Observation Instrument

The observation instrument used to code social behaviors was based on an adaptation of an observational scale (Bauminger, 2002; see Table 1) that had previously been used to evaluate social competence groups for adolescents with ASD (McMahon et al., 2013). We adapted the scale in order to make it more adjusted to our clinical reality. Each category was described in a more specific manner so that observers could understand them better and increase inter-observer reliability. We also eliminated the Negative Interaction dimension, because we rarely observed this type of behavior. Finally, we decided to add a dimension that was very interesting and a fundamental part of communication between our participants: gestures. It is worth noting that gestures are more varied and common in Mediterranean cultures than in northern Europe or the United States.

**Table 1:** Dimensions and category systems in the observation instrument for patients (adaptation of Bauminger, 2002).

Response Levels	Dimensions	Category systems (codes)
Social Initiation (IS): The child begins a new social sequence, distinguished from a previous sequence by a change in activity. Social Response (RS): The child responds verbally and/or non-verbally to social stimuli directed toward him/her by peers.	High positive interaction (HPI): The child exhibits verbal and non-verbal social behaviors that lead to effective social process with peers. Behaviors that serve to start or maintain social interaction.	Eye Contact (CO): The child looks into the eyes of another child. Smile (SON): The child smiles at other children. Affection (AFEC): The child expresses affection for another child, either verbally (e.g., “You’re nice,” “I like you”) or non-verbally (e.g., hugs, touches). Sharing objects (COMOBJ): The child offers his/her objects to another child or shares an object with another child. Sharing experience (COMEXP): The child tells peers about an experience or asks them about their experiences (e.g., “What did you do over the weekend?”). Verbal social communication (COSOVER): The child approaches another child with a social (rather then functional) intention (e.g., “Let’s play”). Talk that reflects an interest in another child (CMI): The child expresses an interest in another child’s hobbies (e.g., “What’s your favorite game/object?”), mood (e.g., “Are you sad?”), etc. Giving help (OFAY): The child offers help to another child.
Social Initiation Social Response	Low level interaction (LPI): The child exhibits behaviors that indicate social intention, but with minimal social enactment, such as close proximity to children without initiating a positive social interaction. Also includes behaviors typical of the autistic syndrome (e.g., echolalia, idiosyncratic language).	Looking: At an action or person without eye contact (MIR): The child looks at the other child’s face or body, or child’s action, without establishing eye contact. Looks to the side, avoiding eye contact (MOL). Close proximity (PX): The child stands in close proximity to another child (3 feet or less) but does not approach the peer. Yes and No (YES/NO): The child only nods his/her head for yes or shakes it for no. Imitation peer (IMIC) or therapist (IMIT): The child imitates the talk or activity of another child or the therapist. Idiosyncratic language (LID): The child uses utterances with no clear meaning. Repetitive behavior (COMREP): The child behaves in a repetitive manner with no clear communication intent, but in close proximity to another child. Functional communication (COMFU): The child approaches or responds to another child with an intention to fulfill his/her own needs, and with no social intention (e.g., “It’s my turn on the computer now”), or just to express something related to the game, without social intent.
Social Initiation Social Response	Gestures	(GECONV): Greet, raise your hand, no / yes (with your head), come, shut up, ok, etc. (GESEN): Gestures that emphasize explanations or participants’ discourse, but that do not add any further meaning. (GEDES): Gestures that indicate the quantity, the size, the form... they describe something. (GESEÑ): Point your hand, arm or finger at something to show it to another. (GEMO): Gestures that indicate an emotion (covering your mouth (surprise or laughter), covering your eyes (disbelief), raising your hands or arm (joy), etc.)

Behavior was grouped into two categories: social initiation (the child/adolescent begins a new social sequence, distinguished from a previous sequence by a change in activity) and social response (the participant responds verbally and/or non-verbally to social stimuli directed toward him/her by peers). Subsequently, each category was organized into three dimensions, depending on the quality of social interaction: high-level positive interaction (HPI; the child exhibits verbal and non-verbal social behaviors that lead to an effective social process with peers and serves to start or maintain social interaction), low level interaction (LPI; the child exhibits behaviors that indicate social intention, but with minimal social enactment, such as close proximity to children without initiating a positive social interaction), and negative interaction (NSI).

These three dimensions involved specific conduct. HPIs included: eye contact, smiling, affection, sharing objects, sharing experience, verbal social communication, talk that reflects an interest in another child’s hobbies and giving help. LPI conduct included: looking, close proximity, “yes” and “no,” imitation, idiosyncratic language, repetitive behavior and functional communication.

As we observed that our sample never codified for negative interaction, we decided to remove this dimension.

### Procedure

#### Data Quality Control Analysis: Inter-Observer Agreement

From the qualitative research perspective, systematic observation was used to obtain data that we managed as a code matrix. Two observers analyzed and coded 14 min of nine group sessions. The degree of inter-observer agreement, calculated with Cohen’s (1988), ranged between 76 and 89%. To obtain this value, 20% of the material was coded and the Kappa coefficient of the A and B sessions (which were randomized) of P3, P5, P6 and P8 participants was calculated. Once we had confirmed the reliability of the data, we codified 14 min during the free play activity of each session to exhaustively record social behaviors throughout the sessions. Finally, we only codified the total material of the participants that appeared in all sessions. For each participant, the nine sessions were organized into three blocks (First block: 1-2-3, second block: 4-5-6 and third block: 7-8-9).

#### Data Analysis

From the quantitative perspective, data were initially analyzed in a descriptive way, showing the corresponding frequencies of the group categories from the ISP and IBN dimensions in three consecutive group sessions through polar coordinate analysis, which is a technique that shows relationships between categories.

To carry out the prospective analysis, the first step is to define a behavior, known as the focal behavior, which, depending on the aims of the study, is believed to generate or trigger a series of connections with other categories, known as conditional behaviors. To detect significant behavioral patterns, it is necessary to compute lag sequential statistics with a focus on positive lags, i.e., events or behaviors that occur *after* the focal behavior. In the case of our study, positive lags identified “forward-occurring” discursive units used by the teachers.

The retrospective, or “backward” perspective, which incorporates what Anguera (1997) referred to as the concept of “genuine retrospectivity,” reveals significant associations between the focal behavior and behaviors that occur *before* this behavior (i.e., negative lags). In this study, this retrospective analysis produced a “mirror-like” image of associations between discursive units that occurred before the focal behavior; the sequence followed was last, second-last, third-last, etc.

As mentioned above, polar coordinate analysis integrates both the prospective and retrospective perspectives, and provides interpretable data through the application of an extremely powerful technique involving the calculation of the *Z*_sum_ statistic, described by Cochran (1954) and later proposed by Sackett (1980). This computation is possible, as both the frequency of the focal behavior (*n*) and the *Z* scores for each of the lags considered are known. These *Z* scores are independent of each other, as they are computed using the binomial test, which compares observed probabilities (corresponding to textual units derived from observation of the therapists’ discourse) with expected probabilities (chance occurrences).

Prospective and retrospective *Z*_sum_ scores can have a positive or negative sign. Each conditional behavior is represented by a vector, which, in turn, is located in one of four quadrants (I, II, III, or IV) depending on the positive or negative sign of the prospective and retrospective Z_sum_ scores. These quadrants indicate whether the focal and conditional behaviors activate or inhibit each other, as follows:

Quadrant I: Mutual excitation between focal and conditional behavior (prospective and retrospective activation).
Quadrant II: Inhibitory focal behavior and excitatory conditional behavior (prospective inhibition and retrospective activation).
Quadrant III: Mutual inhibition between focal and conditional behavior (prospective and retrospective inhibition).
Quadrant IV: Excitatory focal behavior and inhibitory conditional behavior (prospective activation and retrospective inhibition).

With polar coordinate analysis technique, it is possible to generate vectors that show the relationship between the focal behavior and each of the conditional behaviors analyzed. Polar coordinate analysis thus constitutes a powerful statistical technique and a robust methodological tool for identifying all possible interrelationships between the variables of interest in a given study. In short, following a complex process of data reduction, polar coordinate analysis generates a highly informative map containing vectors showing the complex network of interrelationships between behaviors that play a central role (focal behaviors) and other, potentially related, behaviors of interest (conditional behaviors). The corresponding calculations can currently be performed in HOISAN v.1.6.3 (Hernández-Mendo et al., 2012). The first step is to calculate the adjusted residual values for lags −5 to +5; these values are then standardized and combined with the Z_sum_ of the prospective (positive) lags and the retrospective (negative) lags to calculate the length and angle of each vector. The vectors connect the focal behavior with the conditional behaviors. Once the vector angles have been calculated, each vector is assigned to one of four quadrants that indicate the type of relationship between the focal and conditional behaviors. All vectors with a length of 1.96 were considered to be significant (*p* < 0.05).

In order to facilitate the analysis and obtain more data for each participant, the sessions were divided in three blocks: block 1 corresponded to sessions 1, 2 and 3; block 2 corresponded to sessions 4, 5 and 6 and block 3 corresponds to sessions 7, 8 and 9.

In our study, within the HPI (positive high level interaction) dimension, polar coordinates were calculated for the three blocks of sessions considering the following categories as focal and conditional behaviors: eye contact (CO), sharing objects (COMOBJ), sharing experiences (COMEXP), verbal communication (COSOVER) and five types of gestures: conventional gestures (GECONV), emotional gestures (GEMO), emphatic gestures (GESEN), descriptive gestures (GEDES) and pointing gestures (GESEÑ). Within the LPI dimension, polar coordinates were calculated considering the following categories as focal and conditional behaviors: affirmation or denial gestures, functional communication, play and five types of gestures (as with HPI). No negative social interaction (NSI) was registered.

## Results

As previously explained, the final sample consisted of 5 participants with diagnosis of ASD. They all had an IQ within the normal range and presented significant difficulties in social communication and social interaction behaviors. However, the degree of impairment in social communication was distinct for each participant as they showed differences in the quality and frequency of social behaviors (Table 2). We also observed differences in their personal patterns. For instance, some participants exhibited more non-verbal communication behaviors (e.g., eye contact, gestures) than verbal communication while other participants showed the opposite patterns (e.g., verbal communication without eye contact).

**Table 2:** The characteristics of the five participants were as follows.

Participant	Age	Verbal comprehension	ADOS (severity)
P1	16	88	6
P2	15	105	7
P3	14	93	DS 15 (module 4)
P4	14	116	7
P5	14	Missing data	6

To describe relationships between social behaviors for each participant, we applied two types of analysis. We analyzed the number of social behaviors in each block of sessions (frequency) and additionally performed polar coordinate analysis. In general, all participants showed fewer LPIs and high variability in high level positive interaction (HPI) behaviors. As described above, polar coordinate analysis offers information on the development of their interactive behaviors. There was no specific pattern that summarized the progress of all participants through the intervention, as each adolescent took a different course. In the sections below, we describe the development of social behaviors for each participant.

Focal behaviors for each participant were chosen according to the frequency with which they appeared in the sessions of each. For example, in Participant 1, functional communication (COMFU) was more frequent than other types of conduct. Therefore this behavior was selected as focal. In contrast, conditional behaviors were those that appeared more sporadically or were less frequent in the interaction.

### Participant 1

**FIGURE 1:**
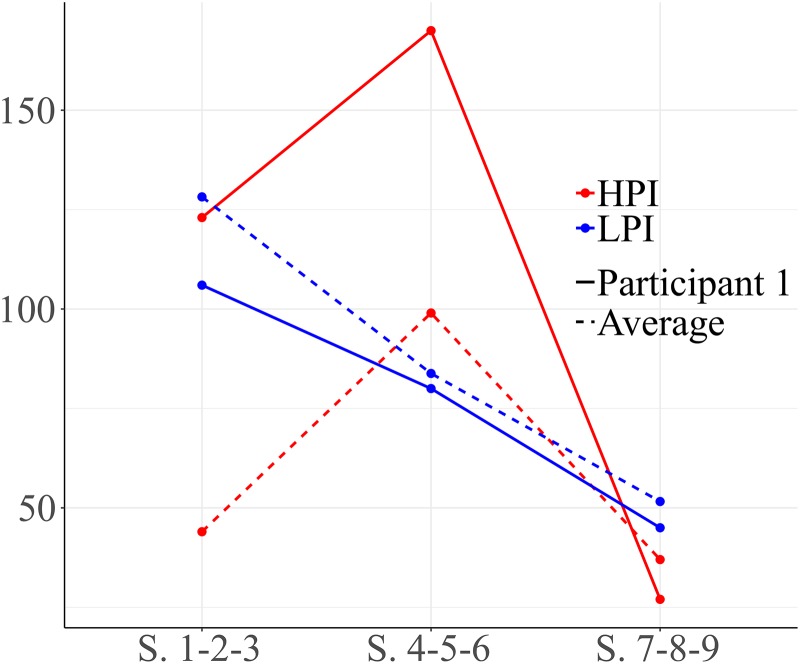
Development of high level positive interaction (HPI) behaviors and low level positive interaction (LPI) behaviors for participant 1. Session blocks 1-2-3 from left to right.

As shown in Figure 1, participant 1 exhibited a high number of HPI behaviors within the first block of sessions (1-2-3). These behaviors increased during the second block, while LPI behaviors decreased. By the end of the intervention, all types of social behaviors decreased.

**FIGURE 2:**
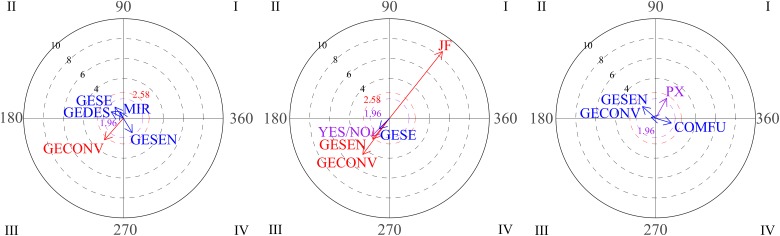
Graphs 1 and 2 represent vectors corresponding to functional communication (COMFU) as the focal behavior during the first and the second blocks of sessions and looking, gestures and functional play as conditional behaviors. Graph 3 represents gestures of nodding/shaking head as focal behavior during the third block and proximity (PX) and functional communication as conditional behaviors.

Polar coordinate analysis was used to analyze relationships between social behaviors. The focal behavior was functional communication. The graphs in Figure 2 show the vectors for the different relationships distributed among the four quadrants through the three blocks of sessions (1-2-3, 4-5-6, 7-8-9). On examining the first block (sessions 1-2-3), it can be seen that functional communication does not stimulate any conduct (quadrant I is empty), whereas vectors located in quadrant IV indicate that functional communication and gestures are mutually inhibited. During sessions 4-5-6 (graph 2), functional communication and functional play activate each other (quadrant I). The third graph represents the gesture of nodding/shaking the head as focal behavior during the third block of sessions (7-8-9). Vectors in quadrant I indicate mutual activation between the gestures of nodding/shaking the head and physical proximity. These results suggest positive development of interactive play during free playtime.

**FIGURE 3:**
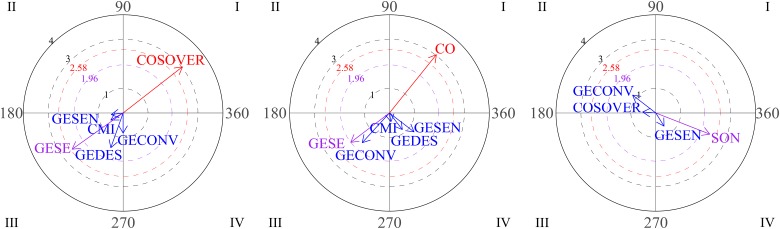
Graph 1 represents vectors corresponding to eye contact (CO) as focal behavior during first block of sessions, and verbal social communication (COSOVER) as conditional behavior. Graph 2 represents verbal social communication as the focal behavior during the first block of sessions. Graph 3 represent vectors corresponding to eye contact as focal behavior during the second and third block of sessions, and conventional gestures and social smile (SON) as conditional behaviors.

The graphs in Figure 3 show positive steps stimulated by eye contact (focal behavior). During the first block (sessions 1-2-3), vectors located in quadrant I indicate that eye contact (CO) and verbal social communication (COSOVER) were mutually activated, which indicates an appropriate strategy. In the second block (sessions 4-5-6), vectors located in the first quadrant indicate mutual activation between eye contact and conventional gestures. Finally, it can be seen that eye contact precedes social smile (SON; quadrant III).

### Participant 2

**FIGURE 4:**
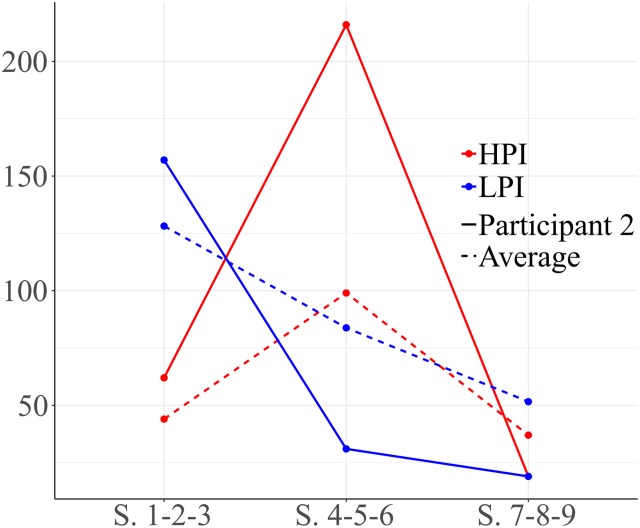
Development of HPI behaviors and low level positive interaction (LPI) behaviors for participant 2. Session blocks 1-2-3 from left to right.

Figure 4 shows the progress of social interaction frequency through the intervention for participant 2. The figure shows that the number of positive interactions increases in the second block with respect to the first block of sessions, while the number of LPIs decreases. Both types of interaction decrease by the end of the intervention.

**FIGURE 5:**
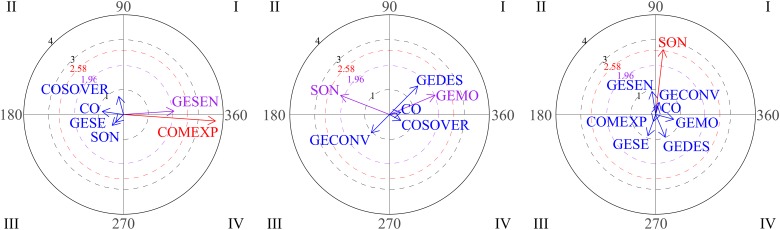
Vectors corresponding to social behaviors of participant 2. Graph 1 and 3 correspond to sessions 4-5-6 and focal behaviors are GEMO and COSOVER. In Graph 2, the focal behavior is GESEN in sessions 1-2-3.

The graphs in Figure 5 represent the development of interactions between social behaviors for participant 2 over the course of the intervention. As shown, gestures are very frequently used by this participant. Block 1 is characterized by mutual activation of emotional gestures and emphatic gestures (quadrant I).

For the second block (sessions 4-5-6), vectors located in quadrant I indicate that verbal social communication (COSOVER) and social smile (SON) were mutually activated. Again, mutual activation was observed between emotional gestures (GEMO) and emphatic gestures (GESEN). No significant result was observed during sessions 7-8-9.

### Participant 3

**FIGURE 6:**
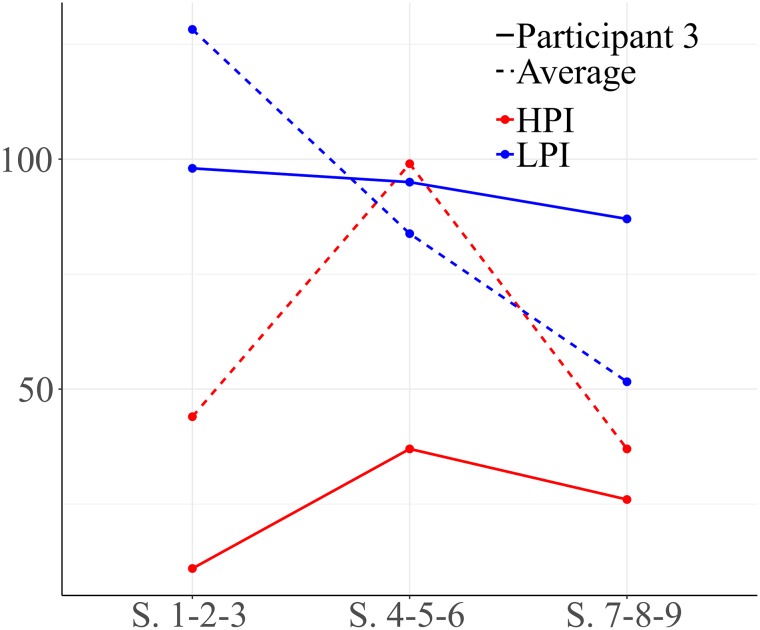
Development of HPI behaviors and low level positive interaction (LPI) behaviors for participant 3. Session blocks 1-2-3 from left to right.

Participant 3 showed a different course to prior participants. As shown in Figure 6, he performed more LPI behaviors than HPI behaviors. High level positive behaviors increased through the intervention, indicating that the quality of his interactions probably improved.

**FIGURE 7:**
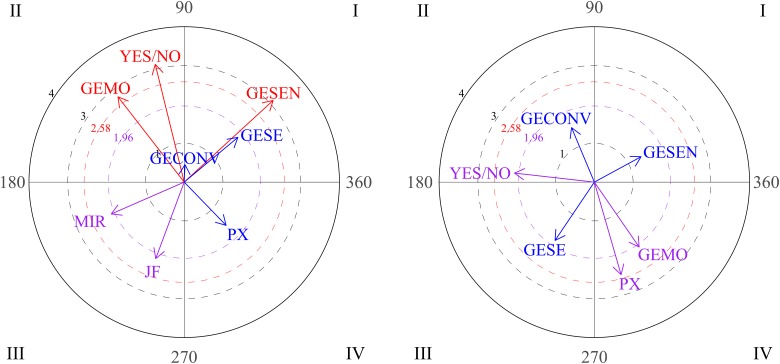
Vectors corresponding to functional communication (COMFU) as focal behavior, and emphatic gestures, gestures of nodding/shaking the head, emotional gestures, looking at an object and functional play as conditional behaviors. Session blocks 1 and 3.

Figure 7 represents interactions between social behaviors for participant 3. The focal behavior was functional communication (COMFU). Significant results are observed for quadrants I and II in the first block of sessions (1-2-3). Vectors located in quadrant I reflect mutual activation between functional communication and emphatic gestures. Vectors situated in quadrant II indicate that functional communication inhibits the gesture of nodding/shaking the head (yes/no), while this gesture activates functional communication. The same pattern is observed with emotional gestures. We observe that looking at an object and functional plays do not stimulate communication.

Vectors for the third block of sessions (sessions 7-8-9) show a change in the relationships between functional communication and other behaviors. In this case, the focal behavior (functional communication) stimulates physical proximity and emotional gestures, but not vice versa (quadrant IV). As in previous sessions, gestures of nodding/shaking the head activate functional communication.

Regarding high level positive behaviors, no significant result was observed in sessions 1-2-3 or 4-5-6. There were significant results in the final sessions (7-8-9). We observed that the social smile activates verbal social communication. This participant showed frequent use of functional communication to initiate and respond in free play, exhibiting more LPI behaviors than high level behaviors (Figure 7). We observed that during the first sessions his functional communication was more often accompanied by gestures. We observed a new component during final sessions: physical proximity to a partner or to a situation. By the end of the intervention, the amount of high level interaction behaviors had increased.

### Participant 4

**FIGURE 8:**
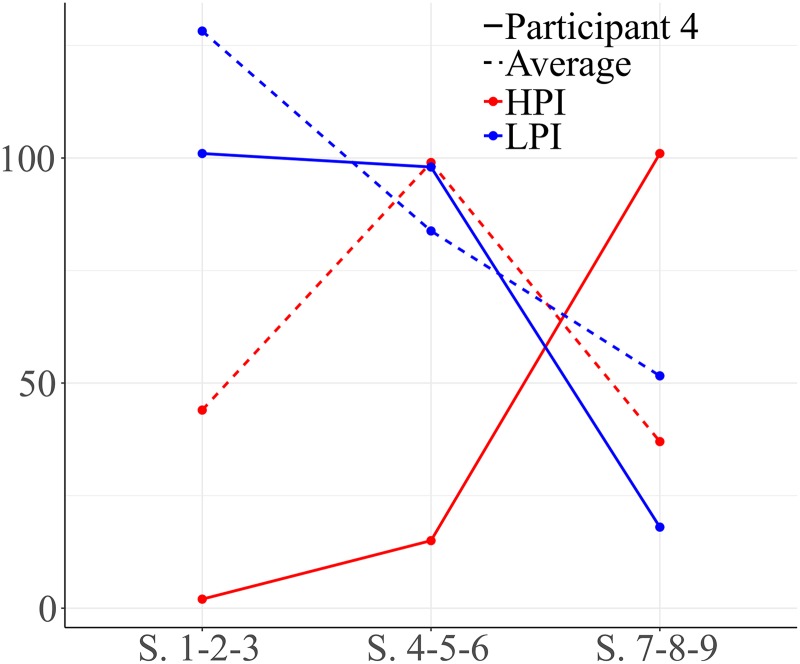
Development of HPI behaviors and low level positive interaction (LPI) behaviors for participant 4. Session blocks 1-2-3 from left to right.

Participant 4 exhibited changes in the use of HPI behaviors and low level behaviors over the course of therapy. Figure 8 shows that during the first block he manifested a high number of LPIs behaviors. However, the amount of high level interaction behaviors increased in the second and third block.

**FIGURE 9:**
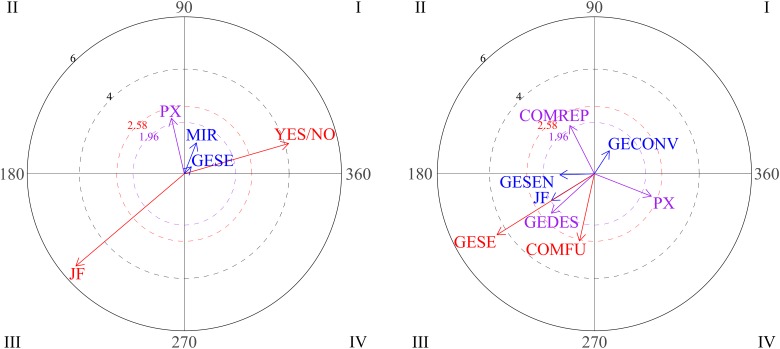
Graph 1 represents vectors corresponding to functional communication (COMFU) as focal behavior and the gestures of head nodding/shaking, physical proximity, functional communication and functional play as conditional behaviors during the first block of sessions (sessions 1-2-3). Graph 2 represents gestures of head nodding/shaking as focal behavior and physical proximity and repetitive behaviors as conditional behaviors during the second block of sessions (sessions 4-5-6).

Graph 1 in Figure 9 shows significant results for vectors located in quadrant I in block 1 (sessions 1-2-3). As can be seen, functional communication and head nodding/shaking (yes/no) gestures are mutually activated. Mutual activation is also observed between physical proximity and functional communication. These observations might offer information about the way this participant initially performs social initiations and social responses. Vectors located in quadrant IV indicate that functional play and functional communication are mutually inhibited.

Results for the second block show interactions among other behaviors (Figure 9, graph 2). Considering head nodding/shaking (yes/no) gestures as focal behavior, it was observed that this behavior activates physical proximity. Repetitive behaviors activate nodding/shaking head gestures.

Head nodding/shaking gestures do not activate emphatic gestures, descriptive gestures or functional communication.

**FIGURE 10:**
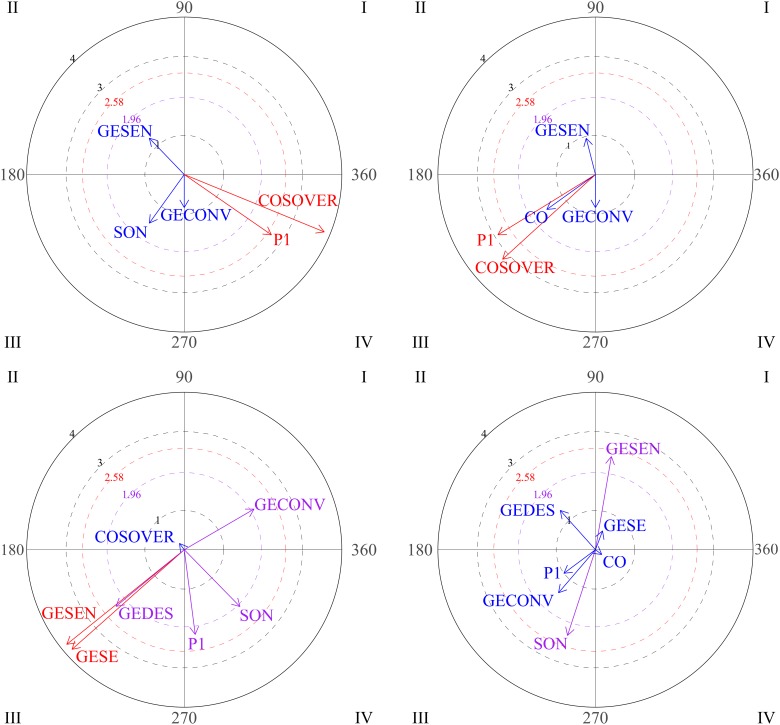
Graph 1 represents eye contact (CO) as focal behavior and verbal social communication (COSOVER) as conditional behavior during the second block of sessions (sessions 4-5-6). Graph 2 represents the social smile (SON) as focal behavior during the second block of sessions (sessions 4-5-6). Graph 3 represents eye contact as focal behavior and social smile, emphatic gestures, descriptive gestures and pointing as conditional behaviors during the third block of sessions (sessions 7-8-9). Graph 4 represents verbal social communication as focal behavior and emphatic gestures as conditional behavior.

Significant results for high level social behaviors are also observed during the second block of sessions (sessions 4-5-6). Considering eye contact as focal behavior, vectors located in quadrant II in the first graph (Figure 10) show that eye contact activates verbal social communication, which is expected in any social interaction. Finally, when the social smile is the focal behavior during this second block, verbal social communication and social smile are mutually inhibited (graph 2, quadrant III).

As can be seen in the third and fourth graphs in Figure 10, this participant shows significant positive results in the final sessions (7-8-9). For the third graph, the focal behavior was eye contact. Quadrant I indicates that eye contact and conventional gestures are mutually activated. Eye contact also activates social smile (graph 3, quadrant IV), which is important in social interactions. Other types of gestures (emphatic, descriptive or pointing gestures) and eye contact are mutually inhibited (graph 3, quadrant III).

The last graph in Figure 10 represents verbal social communication as focal behavior, which activates emphatic gestures.

In general, this participant showed high inhibition during early sessions and his interactions were mainly functional and not socially oriented. He demonstrated positive development and an improvement in the quality of his interactions, probably indicating more interest in social communication.

### Participant 5

**FIGURE 11:**
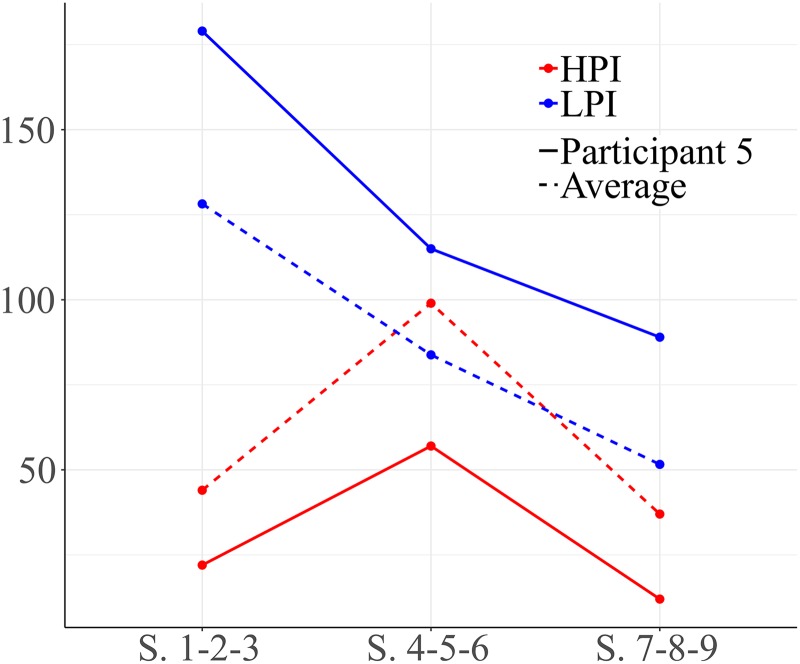
Development of HPI behaviors and low level positive interaction (LPI) behaviors for participant 5. Session blocks 1-2-3 from left to right.

**FIGURE 12:**
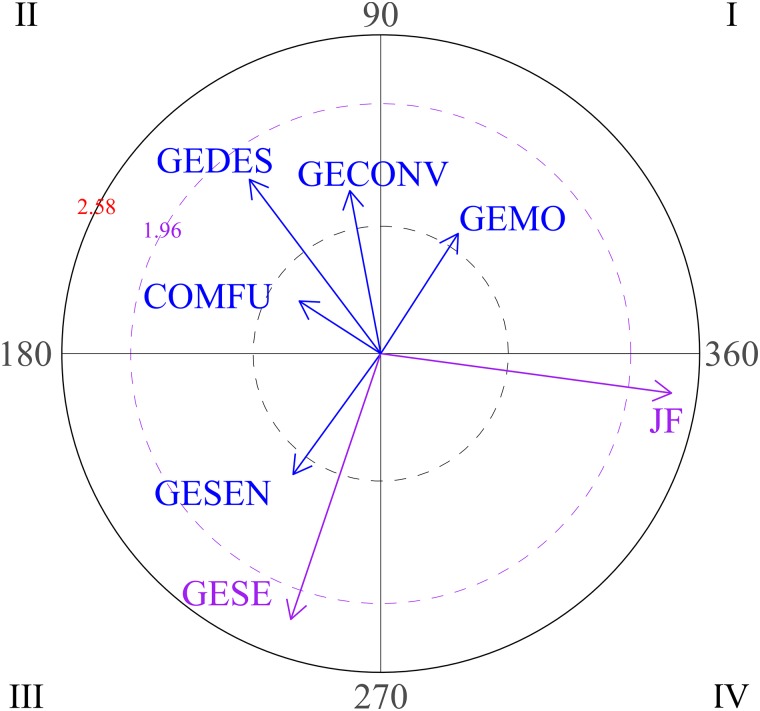
Vectors corresponding to head nodding/shaking (yes/no) gestures as focal behavior, and functional play and pointing as conditional behaviors during the first block of sessions (sessions 1-2-3).

Figure 11 shows the changes of participant 5, manifesting the positive evolution in second block of sessions and decreasing in the final sessions. Figure 12 represents vectors corresponding to head nodding/shaking (yes/no) gestures as focal behavior during the first block of sessions for participant 5. It can be seen that this type of gesture activates functional play (quadrant IV). Vectors located in quadrant III indicate mutual inhibition between head nodding/shaking gestures and pointing.

**FIGURE 13:**
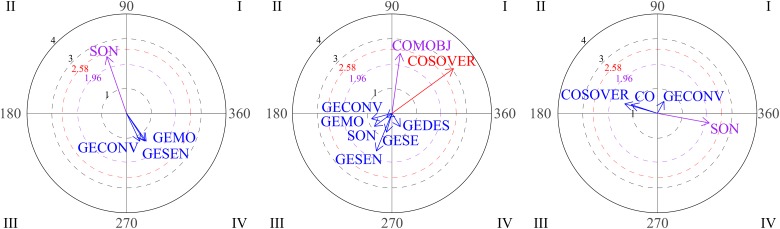
Vectors represent verbal social communication (COSOVER) as focal behavior and social smile as conditional behavior during the first block of sessions (sessions 1-2-3). Graph 2 represents eye contact (CO) as focal behavior and sharing objects and verbal social communication as conditional behaviors during the second block of sessions (sessions 4-5-6). Graph 3 represents emotional gestures as focal behavior and social smile as conditional behavior during the final block of sessions (sessions 7-8-9).

The graphs in Figure 13 show the vectors for the different relationships between social behaviors through the three blocks of sessions for participant 5. During early sessions (graph 1), vectors located in quadrant I indicate that social smile activates verbal social communication retrospectively.

Graph 2 represents eye contact as focal behavior during the second block of sessions (sessions 4-5-6). Vectors situated in quadrant I show mutual activation between eye contact and sharing objects, and between eye contact and verbal social communication. These relationships reflect appropriate use of social communication. Finally, graph 3 shows that emotional gesture activates social smile (quadrant IV).

Participant 5 shows high variability in the use of types of social interactions. He uses both high and low level social behaviors at the beginning and end of the intervention. It can be observed that this participant is able to use HPI behaviors, integrating conduct such as the social smile, sharing objects, eye contact, social communication and emotional gestures. Therefore, although the use of high level interactions is variable, this participant shows good abilities in high level social interaction behaviors.

The results show that there is considerable variability between participants, showing different courses through the sessions, improving or decreasing the number and the quality of their social behaviors. In addition, we observed that the average of HPI in the first block of sessions (1-2-3) was very low, in the second block (sessions 4-5-6) increase the number of HPI behaviors increased, and finally we observed another decrease (sessions 7-8-9).

## Discussion

The main goal of this study was to analyze patterns of social behaviors in a group of adolescents with ASD that participated in a group social-competence intervention program. To evaluate these patterns, we adapted a previously used observation instrument and used polar coordinate analysis, based on an integrative qualitative-quantitative perspective.

The observation instrument presented could be considered as a new tool for coding and analyzing social behaviors. Our final instrument was an adaptation of an observation scale developed by Bauminger (2002), which had already proved its utility in analysis of social behaviors through social-competence group interventions (McMahon et al., 2013). With this study, we also observed that this instrument could be useful for polar coordinate analysis. The reliability results in the data quality control analysis support the adequacy of the data obtained. This observation instrument might have future applications in the field of psychotherapy.

Regarding polar coordinate analysis, we show the potential of the application of this observational methodology in analyzing behaviors in a group of adolescents with ASD. As has already been noted, Portell et al. (2015) described observational methodology as the type of evaluation that allows analysis of spontaneous behaviors in psychotherapy. Our results show that participants exhibited highly variable patterns of social behavior and different courses of development. Therefore, it is not possible to summarize their behaviors in one single pattern that represents the whole group of adolescents. Nevertheless, we were able to observe spontaneous behavior in a semi-structured social environment, which allowed us to observe variation in behaviors for each participant.

In common with Anguera et al. (2018), we established that the use of observational methodology offers the opportunity to obtain a large amount of data that can be analyzed with different techniques. These data offer a great variety of options when analyzing behaviors in a more sensitive and detailed manner, offering information to which questionnaires are not sensitive (Kasari et al., 2014).

Similarly, Castellano and Hernández (2003) suggest that polar coordinate analysis offers the chance to observe unique qualitative details which can be subsequently transformed into quantitative data to be interpreted in a more global manner. In our study, this type of analysis offered information about bidirectional relationships that appear between the behaviors of each participant. In the field of ASD, obtaining detailed information about behaviors would be very useful for therapists, as it can provide information on the way one type of behavior might activate or inhibit another. In our study, we obtained information on how one behavior activated or inhibited another in the same person. Another type of analysis could examine interactions among different participants. For example, how a therapist activates or inhibits other participants’ behaviors could be explored (Arias-Pujol and Anguera, 2017).

It is also valuable to know the type of behaviors that a person tends to exhibit. For instance, knowing whether a participant tends to display more low or high level behaviors could be important. Therapists may be interested in observing whether this tendency changes through the course of their interventions.

Similar observational methodology and polar coordinate analysis has been used to analyze behaviors in sports competitions, such as soccer (Maneiro and Jiménez, 2018) and handball (Morillo et al., 2017). These studies support the numerous possible applications of this type of methodology.

## Conclusion and Future Lines of Research Work

We observed that each participant took a unique course, increasing or decreasing the number and quality of their social behaviors. In accordance with the literature, we observed some increment in the amount of appropriate social conduct. We cannot generalize to a pattern of progress but can say that there were changes and differences between the beginning and the end of the intervention. Therefore, we consider that observational methodology might be useful in the field of psychotherapy and ASD, offering information about changes and development that cannot be obtained with other traditional measures, such as questionnaires.

For future lines of research, it would be interesting to correlate the different variables of the initial evaluation (i.e., ADOS2, WISC-V) with data obtained from polar coordinate analysis. This would provide more information about the possible changes in the degree of severity or difficulties related to ASD. Furthermore, studies with a greater number of sessions are needed to obtain more data that support these findings. In addition, it is vital to conduct more studies that include observational methodology and mixed methods analysis to obtain more evidence on the real utility of this methodology. It is important to obtain reliable data that support this type of analysis, which would, in turn, allow researchers to obtain detailed information on spontaneous behaviors and then transform this information into quantitative data. Professionals in the field of ASD need new methods to evaluate their interventions and the changes in their patients. Each little change might be important in a child’s development.

## Ethics Statement

This study was carried out in accordance with the recommendations of Spanish Official College of Psychologists General Council’s Ethical Code with written informed consent from all subjects. All subjects gave written informed consent in accordance with the Declaration of Helsinki. The protocol was approved by the Research Committee of Hospital Sant Joan de Déu.

## Author Contributions

MÁM and CA developed the project, codifying the videos, running the group therapy and writing the manuscript. EA-P contributed to documenting, drafting and writing the manuscript and gave her approval to the final version to be published. MTA wrote the methods section and performed the polar coordinate analysis. MM, NE, MD-J, GB, MG-R, and JR-M contributed to documenting, drafting and writing the manuscript. JR-M designed all the figures of the manuscript.

## Conflict of Interest Statement

The authors declare that the research was conducted in the absence of any commercial or financial relationships that could be construed as a potential conflict of interest.
